# Postinfectious Bronchiolitis Obliterans in Children

**DOI:** 10.1155/carj/7790381

**Published:** 2025-07-07

**Authors:** Linfang Wan, Zhonghong Wang

**Affiliations:** Department of Pediatrics, First People's Hospital of Jingzhou, Jingzhou, Hubei, China

**Keywords:** adenovirus pneumonia, bronchiolitis, children, *Mycoplasma pneumoniae* pneumonia

## Abstract

Bronchiolitis obliterans is a rare form of chronic obstructive lung disease, with postinfectious bronchiolitis obliterans being particularly prevalent in pediatric populations. The pathogenic investigations of pediatric postinfectious bronchiolitis obliterans predominantly center on adenovirus and *Mycoplasma pneumoniae*. Following the illness, children's lung function is impacted to varying extents; however, a definitive diagnosis relies on lung biopsy, which is not conducive to early detection and timely intervention. Consequently, there remains a need for novel research methodologies. This article synthesizes the 2023 China Reformulation of the Expert Consensus on the Diagnosis and Treatment of Bronchiolitis Obliterans in Children along with recent studies, providing a comprehensive overview of early diagnosis, treatment modalities, and preventive strategies for postinfectious bronchiolitis obliterans in children. Additionally, it outlines future research directions aimed at enhancing pediatricians' understanding of this complex disease.

## 1. Introduction

Postinfectious bronchiolitis obliterans (PIBO) is a rare lung disease characterized by chronic airflow limitation, resulting from an infection that damages the lower airways and triggers an inflammatory and fibrotic response, ultimately leading to partial or complete obstruction of the airway lumen with low response to bronchodilators [1–3].

It should be noted that PIBO is a sequel that requires supportive treatment. This article proposes treatment approaches grounded in a single study rather than those derived from controlled comparative studies. In addition to infectious bronchiolitis obliterans (BO), other etiologies of BO in children include the bronchiolitis obliterans syndrome (BOS) following stem cell and lung transplantation. These latter conditions, however, are beyond the scope of our current discussion.

## 2. Etiology

The predominant etiological agent of PIBO in children is adenovirus, which has been extensively investigated. The most frequently identified subtypes are types 3, 7, 11, and 21, accounting for 20%–69% of PIBO cases [4, 5]. Additionally, Adenovirus-55 has also been reported [6]. *Mycoplasma pneumoniae* is more prevalent in older children and has emerged as a significant cause of PIBO in recent years, particularly with the rise of *Mycoplasma pneumoniae* pneumonia in Asia. Respiratory syncytial virus (RSV), which peaks during winter months, is another recognized cause of PIBO. Other pathogens, including measles virus, influenza virus, parainfluenza virus, herpes simplex virus, *Bordetella pertussis*, *Mycobacterium tuberculosis*, *Chlamydophila pneumoniae*, *Legionella* species, and SARS-CoV-2, can also contribute to PIBO [1, 7–12]. The distribution of these pathogens is summarized in Table 1. Notably, infections caused by these pathogens may occur individually or concurrently, contributing to the development of PIBO.

## 3. Epidemiology

PIBO arises from a severe lower respiratory tract infection, which can manifest across all age groups. Cases of PIBO following infections with adenoviruses and other pathogens have been documented globally, influenced by various environmental exposures and genetic predispositions.

The precise incidence and prevalence of PIBO remain undetermined due to the limited sample sizes and geographic variability in existing studies, resulting in a lack of comprehensive epidemiological data.

There are notable gender and age disparities in the incidence of PIBO, with several studies indicating that approximately two-thirds of affected children are male, and the typical age of onset is under 2 years [4, 13]. The disease predominantly occurs in the Southern Hemisphere (e.g., Argentina [5], Chile [14], Southern Brazil [15], and New Zealand [16]), but it is also observed in the Northern Hemisphere (e.g., Turkey [17], Russia [18], and Canada [19]). Furthermore, PIBO appears to be more prevalent among specific populations such as Argentines, Native Americans, and Native Koreans—suggesting a significant genetic component to its etiology [20–23]. Recently, there has been a rising number of PIBO cases reported in Asia, particularly in China and Korea [24–28].

## 4. Pathology

### 4.1. Pathological Features

PIBO is predominantly characterized by neutrophilic inflammation in the small bronchioles, marked by elevated levels of proinflammatory cytokines. This inflammatory response leads to tissue remodeling and fibrosis within the small airways, ultimately resulting in airway narrowing or complete obstruction [29]. These pathological changes are typically initiated by a viral lesion assault following an acute lower respiratory tract infection [29]. Tissue injury and abnormal repair mechanisms lead to damage of small airway epithelial cells and subepithelial structures, triggering a progressive inflammatory response that induces BO. This process results in remodeling of lower airway tissues, small airway fibrosis, and airway obstruction, ultimately culminating in partial or complete obstruction of the terminal bronchioles or respiratory bronchiolar lumens—hallmarks of BO pathology [5]. BO is classified into two categories: proliferative and constrictive. Proliferative BO involves lumen obstruction due to granulation tissue and polyps within the fine bronchial tubes, while constrictive BO is characterized by peribronchial fibrosis leading to partial or complete narrowing of the lumen [5]. Research has demonstrated that the majority of children exhibit constrictive BO, although both types of pathological changes can coexist [30]. Constrictive BO typically exhibits poor responsiveness to corticosteroid therapy.

Lower respiratory tract infections and various injuries can lead to dysfunction or localized necrosis of small airway epithelial cells, impaired regeneration of these cells, accumulation of fibrin exudate within the lumen, fibroblast infiltration, destruction of airway basal cells, collagen deposition, and vascular hyperplasia. These pathological changes culminate in severe airway obstruction and varying degrees of deformity in the airway walls following subsequent scarring [30, 31]. In addition to involvement of small airways, medium and large airways may also exhibit similar lesions. Furthermore, bronchiectasis may develop in more severely affected airways.

### 4.2. Immunology and Biomarkers

The immune mechanisms underlying PIBO remain under investigation. The respiratory epithelium, which is compromised by viral infection, serves as the interface between the host and the environment. A growing body of evidence indicates that this tissue plays a crucial role in balancing repair processes and inflammatory responses [32]. Numerous respiratory epithelial attachment receptors have been extensively studied, including coxsackievirus and adenovirus receptors (CARs), CD46, glycan GD1a, polysialic acid, and desmoglein-2 [33]. These receptors influence basal permeability and barrier integrity, resulting in the release of proinflammatory cytokines, neutrophil migration, and peribronchial inflammation [34]. Consequently, the impairment of the epithelial immune response due to viral infection may regulate the secretion of inflammatory mediators and subsequent tissue remodeling, potentially contributing to the pathogenesis of PIBO [29]. Inflammatory activation may persist for several years following acute infection and could contribute to the fibrotic processes associated with PIBO [35]. Additionally, a range of biomarkers may be implicated in this pathophysiological process, as summarized in Table 2.

Patients with PIBO exhibit elevated levels of serum and/or alveolar lavage/sputum neutrophils, as well as increased concentrations of interleukins (ILs) 1β, 6, 8, YKL-40, Caspase-1, transforming growth factor-beta (TGF-β), Postn, INF-γ, β2-defensin, and cathelicidin (LL-37). Conversely, decreased levels of IL-18, IL-27, KL-6, miR-335-5p, miR-186-5p, miR-30b-5p, and miR-30c-5p have also been observed [17, 36–46]. These findings suggest that these cells; cytokines; chemokines; and profibrotic factors may play a significant role in the pathogenesis of PIBO.

## 5. Clinical Findings

PIBO does not present with specific symptoms. However, a diagnosis should be considered in an otherwise healthy child who develops shortness of breath, cough, wheezing, exercise intolerance, and hypoxemia persisting for at least 6 weeks following a severe acute lower respiratory tract infection. The severity of symptoms can vary; in severe cases, these symptoms may persist and can lead to death from respiratory failure [30, 31]. Initial mild symptoms do not preclude the development of persistent dyspnea as the condition progresses, which is more commonly observed in infancy. In older children, symptoms of PIBO may be easily mistaken for asthma or other lung-specific diseases. Symptoms such as cough, wheezing, and dyspnea may improve as the lungs develop [47].

## 6. Diagnostic Workup

### 6.1. Arterial Blood Gas Analysis

This assessment is utilized to evaluate the severity of the child's condition and may indicate hypoxemia during acute exacerbations or in critically ill pediatric patients. The acute phase of arterial oxygen saturation (SaO_2_) can be markedly decreased, with gradual improvement observed over several years [48].

### 6.2. Lung Function Testing

This assessment can aid in the diagnosis of children with PIBO aged 4–6 years and provide insights into the severity and progression of the disease. Teper et al. reported in their study that infants with chronic lung disease following severe adenovirus infection exhibited significant alterations in respiratory function, including changes in peak tidal expiratory flow (PTEF), PTEF/tidal volume ratio, and other parameters. Additionally, these infants demonstrated reduced respiratory system compliance and increased resistance, characterized by severe obstructive lung function deficits and diminished pulmonary dilation. Notably, there was no observed response to inhaled bronchodilator administration [48]. This study established a foundational framework for the investigation of lung function in PIBO patients. Children with PIBO are characterized by irreversible airway obstruction, as evidenced by a reduced forced expiratory volume in one second (FEV1). This condition is further indicated by a decrease in the Tiffeneau ratio (FEV1/forced vital capacity (FVC)), reflecting diminished FEV1 relative to FVC [35, 49]. Additionally, reductions in peak expiratory flow (PEF) and significant decreases in forced expiratory flow at midrange lung volumes (FEF25-75) have been observed [30, 31, 50]. Children with PIBO exhibit increased residual air volumes and total residual ratios due to hyperinflation and air trapping [35]. Small infants undergoing volumetric assessments demonstrate a marked increase in lung volume and airway resistance; this elevated residual air volume correlates with signs of air trapping and manifestations of hyperventilation on high-resolution computed tomography (HRCT) [2].

Long-term follow-up studies indicate that children with PIBO experience persistent obstructive ventilatory deficits in lung function, with a subset potentially exhibiting restrictive or mixed ventilatory dysfunction [4]. Although the measured values of FEV1 and FVC may show annual increases, improvements in the FEV1/FVC ratio are not significant, suggesting an imbalance in the development of airways and lung parenchyma among children with PIBO [49].

Lung function assessments can also serve as a differential diagnostic tool. Airway reversibility is observed to be lower in children with PIBO compared to those with asthma, both before and after the administration of bronchodilators. While it was previously believed that the bronchodilator response in PIBO was predominantly negative, recent studies have demonstrated that bronchial dilation tests in some children with PIBO have yielded positive results [28, 51–53]. Although PIBO does not affect the entire lung, it impacts all bronchioles. Consequently, those less affected may exhibit hyperreactivity and demonstrate a degree of bronchodilator response in spirometry [15, 51]. This hyperreactivity could be due to a genetic determination or be secondary to PIBO. Nonetheless, due to the limited number of cases examined, these findings may not fully represent the true situation.

Given the limitations of routine lung function assessments in detecting early lesions of PIBO, recent years have seen the emergence of the inert gas washout test as a sensitive and feasible tool for identifying early small airway damage in both children and adults with chronic lung disease. The lung clearance index (LCI), measured via sulfur hexafluoride multiple breath washout (MBW) testing, serves as a valuable supplementary tool for diagnosing and evaluating PIBO in infants and young children [30, 54]. A prospective study conducted by Kim et al. evaluated the utility of LCI in assessing the extent of small airway obstruction in pediatric patients with PIBO, serving as a supplementary tool for the diagnosis and evaluation of PIBO in infants and young children [55]. Due to the significant obstructive component that these children present, the determination of LCI becomes extremely prolonged and requires a more extensive set of experimental data to validate their applicability.

Forced oscillation technique (FOT) and impulse oscillation technique (IOS) offer distinct advantages over traditional spirometry in the pediatric population, as they do not require specific breathing maneuvers and are more suitable for assessing respiratory impedance in younger preschool-aged patients. However, there is a lack of studies investigating the feasibility of FOT and IOS in PIBO [56, 57].

### 6.3. Imaging

PIBO air trapping may not be evident in the initial stages of infection; however, as the disease progresses, nonspecific manifestations such as overinflation and thickening of the airway wall, including linear or reticular opacities, become observable. In advanced stages, some children may present with unilateral hyperlucent lung, characterized by increased translucency, texture thinning, and reduced volume in one or more lobes of the affected lung [58].

HRCT is the primary noninvasive modality for confirming the diagnosis of PIBO and plays a crucial role in diagnosis, differential diagnosis, condition assessment, and follow-up [4, 13]. Common HRCT findings associated with PIBO include mosaic perfusion patterns, air trapping, bronchial wall thickening, lung atelectasis, and bronchiectasis; however, the prevalence of these imaging manifestations has varied across different studies [4, 13, 59]. In children with a typical clinical history, the presence of mosaic perfusion signs on HRCT serves as the strongest predictor of PIBO. The expiratory phase is more sensitive than the inspiratory phase for diagnosing small airway obstruction due to clearer visualization of air trapping signs and a higher incidence of mosaic perfusion patterns. However, expiratory phase scanning may be challenging in younger children; thus, reliance on air trapping signs can limit diagnostic accuracy in this population. Kim et al. demonstrated that airway measurements (WA) and air trapping assessments (ATV) from lung computed tomography (CT) can serve as valuable tools for evaluating disease severity in children with PIBO [60].

Magnetic resonance imaging (MRI) has also been employed in the assessment of PIBO. Recent advancements in MRI sequences have demonstrated the capability to produce high-quality images across a variety of lung lesions; however, their clinical application remains limited due to factors such as medication interactions, equipment availability, and age-related constraints [61].

### 6.4. Lung Biopsy

Lung tissue obtained through biopsy that exhibits characteristic airway lesions is regarded as the gold standard for diagnosing PIBO [30]. However, lung biopsy presents significant clinical challenges due to its invasive nature, which may exacerbate the patient's condition, and the heterogeneous distribution of lesions that can result in sampling missed. Studies have indicated that lung biopsy specimens may be normal or nondiagnostic in up to one-third of pediatric cases [62].

### 6.5. Bronchoscopy

Bronchoscopy with bronchoscopic alveolar lavage (BAL) is commonly employed to evaluate PIBO, although it cannot definitively confirm the diagnosis. This procedure can distinguish between other types of chronic airway obstructive diseases and detect airway deformities, such as tenderness; however, it does not address persistent infections caused by viral, fungal, or bacterial pathogens.

### 6.6. Six-Minute Walk Test (6MWT) and Cardiopulmonary Exercise Test (CPET)

Both methods reflect the degree of exercise intolerance in patients with lung disease, are appropriate for school-age children and older, and can serve as assessments of exercise capacity [63].

### 6.7. Nocturnal Polysomnography (PSG)

It can be utilized to evaluate sleep quality in children with chronic lung disease [64].

## 7. Risk Factor

### 7.1. Pathogenic Factor

Infections caused by adenovirus and/or *Mycoplasma pneumoniae* are significant risk factors for the development of PIBO [5, 8, 29].

The incidence of PIBO following adenovirus pneumonia and *Mycoplasma pneumoniae* pneumonia remains unclear. A study involving 187 cases of adenovirus pneumonia from Jilin, China, reported that the incidence of severe adenovirus pneumonia was 66 out of 187 (35.3%), with 20 cases (30.3%) progressing to PIBO [26]. In a separate investigation on adenovirus-induced PIBO, a significant increase in lactate dehydrogenase (LDH) levels was observed in affected children [65]. Elevated LDH levels and pleural effusion are considered risk factors for the progression from *Mycoplasma pneumoniae* infection to PIBO [65, 66]. E. Lee and Y. Lee demonstrated that respiratory viral coinfections (including those caused by adenoviruses), poor response to treatment for *Mycoplasma pneumoniae* pneumonia, and elevated serum LDH levels were associated with an increased risk of developing PIBO after *Mycoplasma pneumoniae* infection; however, macrolide resistance in *Mycoplasma* was not identified as a risk factor [27].

### 7.2. Severity of Disease

Risk factors for the development of PIBO include hypoxemia, a history of mechanical ventilation, hospitalization exceeding 30 days, multifocal pneumonia, hypercapnia, shortness of breath, and prolonged fever duration [4, 25, 65–69]. A study has indicated that children exhibiting persistent wheezing or acute respiratory failure during the acute phase of severe adenovirus pneumonia are at increased risk for developing PIBO as an independent factor [26].

### 7.3. Genetic Factors

Mannose-binding lectin (MBL) deficiency is more prevalent in children with PIBO compared to healthy controls, and this genetic trait is significantly associated with the severity of the acute phase of the disease as well as the development of PIBO [70]. MBL insufficiency has been shown to predispose individuals to severe respiratory viral infections, including RSV, influenza viruses, and adenoviruses [71, 72].

## 8. Diagnostic Criteria

Histopathological examination of lung biopsy specimens is considered the gold standard for diagnosing PIBO, which is characterized by damage and inflammation of small airway epithelial cells and subepithelial structures, leading to excessive fibrous proliferation that results in partial or complete obstruction of the lumens of terminal and respiratory bronchi.

In clinical practice, the diagnosis of PIBO is typically based on a combination of patient history, clinical manifestations, HRCT, and ancillary tests such as pulmonary function assessments. PIBO can be diagnosed clinically in the absence of histopathological confirmation [73].

Diagnostic criteria for PIBO:

1. A history of relatively severe prior lower respiratory tract infections, particularly those caused by adenovirus, *Mycoplasma pneumoniae*, and measles virus.
2. Persistent or recurrent signs and symptoms of airway obstruction are characterized by clinical manifestations such as ongoing wheezing or coughing, shortness of breath, dyspnea, and exercise intolerance following pulmonary infection. Wheezing and wet rales were consistently audible at the site of involvement, persisting for more than 6 weeks and demonstrating a poor response to bronchodilator therapy.
3. Lung function tests indicated obstructive ventilatory dysfunction or mixed ventilatory dysfunction, characterized by FEV1/FVC ratios of less than 80% or predicted FEV1 values below 80%, with bronchodilator responses predominantly negative.
4. HRCT imaging of the lungs revealed mosaic perfusion patterns and air trapping, along with bronchial wall thickening, bronchial dilation, and other associated findings.
5. Exclude other chronic lung diseases, including bronchial asthma, various congenital bronchopulmonary developmental anomalies, tuberculosis, diffuse panbronchiolitis, and cystic fibrosis (CF).

It is important to note that the early diagnosis of PIBO is challenging, and its diagnosis may significantly lag behind clinical presentation. Reported experiences indicate that the time from symptom onset to PIBO diagnosis ranges from 15.4 to 87.6 months [22, 73, 74]. Delayed diagnosis can lead to more severe complications. To facilitate the early identification of at-risk patients, Colom and Teper developed a clinical tool for predicting PIBO [5, 75], as illustrated in Table 3. This tool comprises three variables: typical clinical history, adenovirus exposure history, and mosaic patterns observed on HRCT. A score of ≥ 7 suggests a potential risk for PIBO. Typical clinical history is defined as a patient who was previously healthy but suffered a severe episode of bronchiolitis, and subsequently developed chronic hypoxemia (SaO_2_ < 92%) for more than 60 days. However, this prediction tool may overlook mild cases of PIBO.

## 9. Treatment and Management

The treatment guidelines and methods for PIBO have not achieved global uniformity. In regions with a high prevalence of this disease, such as Argentina and Chile, national guidelines for PIBO have been established [76, 77]. Inhaled glucocorticoids, montelukast, and low-dose azithromycin are recommended for children exhibiting clinical symptoms such as wheezing and dyspnea. The addition of nebulized inhaled bronchodilators at the onset of the disease or during acute exacerbations may alleviate symptoms [78, 79], while systemic glucocorticosteroids may be indicated in severe cases. Based on the latest BO guideline of China, it is advisable that children with PIBO undergo regular follow-up assessments, including periodic reviews of HRCT and pulmonary function tests every 3–6 months. As the disease progresses, the interval for HRCT evaluations can be extended to every 6 months to 1 year [1]. Considering patient safety, other guidelines recommend limiting the frequency of CT scans [76]. Treatment regimens should be adjusted according to changes in disease status and therapeutic response.

### 9.1. Glucocorticosteroids

The most prevalent anti-inflammatory treatment is glucocorticoid therapy. However, there has been no standardization regarding the route, method, and timing of administration. The consensus in the field can be summarized as follows.

Systemic glucocorticosteroids should be administered in severe cases or early in the disease course [5, 80], and their use should be discontinued promptly if there is no response to treatment or if significant adverse reactions occur (e.g., immunosuppression, osteoporosis, and growth retardation). These medications can be used in conjunction with inhaled corticosteroids. Oral prednisone or methylprednisolone tablets may be prescribed at a dosage of 1-2 mg/(kg·day), with full dosing for 2 weeks to 1 month, followed by a gradual reduction; maintain the minimum dosage for a period of 3–6 months [1, 13]. In cases of early PIBO or cute exacerbations characterized by wheezing, intravenous administration of methylprednisolone at a dose of 2 mg/(kg·day) may be considered, given once or twice daily until the condition stabilizes enough for oral administration [1]. For critically ill children with PIBO, intravenous pulse therapy using methylprednisolone can be administered for three consecutive days each month at doses ranging from 10 to 30 mg/(kg·day), not exceeding a maximum daily intake of 1 g for a duration of 3–6 months [1, 76]. Literature reports indicate significant improvements in oxygen saturation and lung function among children with BOS and PIBO following high-dose methylprednisolone pulse therapy after bone marrow transplantation [81].

Glucocorticosteroids may be administered via inhalation if clinical symptoms are mild and stable, or as maintenance therapy for systemic hormone application [1, 13]. The recommended dosage is 0.5–1.0 mg/day, administered twice daily using a nebulized solution of budesonide (1 mg/2 mL) with jet nebulizers, which are suitable for children of all ages [1]. Other inhalation devices can be selected based on age, including fluticasone propionate aerosol with mist storage canister, budesonide-formoterol inhaler, and salmeterol-triamcinolone inhaler [1]. Dosage adjustments should be evaluated every 3 months, with a minimum treatment duration of at least 1 year or longer [78, 82].

A study has demonstrated that children with PIBO may respond favorably to glucocorticoid pulse therapy when pretreatment CT indicates bronchial wall thickening [80]. Prior to the onset of airway fibrosis, glucocorticoid administration is effective in managing acute exacerbations of PIBO by enhancing lung function and exercise capacity; however, the long-term benefits remain largely unknown [30]. Some studies investigating long-term glucocorticoid pulse therapy (methylprednisolone at 30 mg/kg/day for 3 days per month over a multi-month period) for PIBO reported reductions in worsening wheezing and hospitalization rates, improvements in oxygen saturation, a favorable safety profile, and transient adverse effects among treated children [83, 84]. Conversely, another study indicated that inhaled glucocorticoids are effective in improving lung function and alleviating airway obstruction—particularly persistent inhaled corticosteroids—in patients older than 5 years with remission of PIBO [51].

### 9.2. Macrolides

The precise mechanism underlying the anti-inflammatory effects of azithromycin remains unclear; however, it is widely recognized that it inhibits neutrophil activity and reduces cytokine secretion (e.g., interleukin-6 (IL-6), IL-8, and TNF), which can lead to significant improvements in lung function among children with posttransplant BOS [83, 85]. Azithromycin has been studied less frequently as a monotherapy in patients with PIBO and is more commonly used in conjunction with glucocorticoids and leukotriene receptor antagonists [24, 78]. It is primarily indicated for children with alveolar lavage neutrophils greater than 0.15; conversely, patients with alveolar lavage neutrophils below this threshold exhibit poor efficacy [86]. Long-term oral administration of macrolides necessitates regular monitoring of liver function [83].

The optimal dosage of azithromycin remains a subject of debate. The 2023 Chinese guidelines for PIBO recommend administering azithromycin at a dose of 5 mg/(kg·day) for 3 days per week, with a treatment duration suggested for up to 6 months [1]. In another study, oral administration of azithromycin was recommended at a dosage of 10 mg/kg/day for 3 days each week [13, 77, 87]. A study conducted by Li et al. involving 42 children diagnosed with PIBO demonstrated some benefits associated with the combination of systemic corticosteroids and oral azithromycin [8].

### 9.3. Leukotriene Receptor Antagonists

The leukotriene receptor antagonist montelukast, which inhibits airway inflammation, has an uncertain efficacy in PIBO. It may be administered to children at standard doses for a recommended duration of 6 months [88].

The combination of budesonide, montelukast, and azithromycin enhances lung function and alleviates respiratory symptoms in patients with PIBO. A study demonstrated that children with PIBO who received this combination therapy exhibited marked improvements in lung function after 3 months of treatment [78]. However, current available evidence and research are insufficient to substantiate this assertion.

### 9.4. Oxygen Therapy and Respiratory Support

Oxygen saturation should be monitored, particularly when it falls below 0.92; in such cases, the child should receive oxygen therapy to achieve a saturation level of 0.94 or higher. Oxygen therapy can be administered at home using an oxygen concentrator. For critically ill patients, continuous positive end-expiratory pressure (CPAP) ventilation or respiratory support via a ventilator may be indicated [1, 76, 77].

### 9.5. Lung Physiotherapy

This approach includes postural drainage, chest percussion, and high-frequency chest wall vibration to facilitate the clearance of respiratory secretions, thereby effectively reducing secretion retention. These techniques aim to decrease sputum volume, improve its characteristics, and assist in the reopening of pulmonary atelectasis. In older children, additional methods such as guided coughing, forceful expiratory techniques, active breathing cycles, spontaneous drainage, and mechanical inhalation–exhalation may also be employed as part of pulmonary rehabilitation therapy [1].

### 9.6. Bronchodilators

Airflow limitation in PIBO is typically regarded as irreversible, exhibiting minimal responsiveness to bronchodilators. However, a recent study has reported a positive bronchodilator response in pediatric patients with PIBO. Further research with larger sample sizes is required to provide robust evidence [22, 51–53]. Short-term inhalation of short-acting β2-adrenergic agonists may partially alleviate wheezing symptoms and enhance lung function [15]. Long-acting β2-adrenergic agonists are not used as monotherapy; rather, they are recommended for use in conjunction with inhaled or systemic corticosteroids, which may help reduce the required dosage of corticosteroids, particularly for patients exhibiting combined wheezing symptoms [1]. These viewpoints still require more evidence to support.

### 9.7. Anti-Infective Drugs

Anti-infective drug therapy is not recommended in the absence of secondary bacterial, viral, or fungal infections; however, it is indicated when patients exhibit signs of infection such as fever, exacerbation of wheezing symptoms, and increased sputum volume [1, 76, 77]. Common pathogens include *Streptococcus pneumoniae*, *Haemophilus influenzae*, *Mycoplasma pneumoniae*, and adenovirus, among others, including mixed infections [89]. For pathogen detection, options include conducting culture, polymerase chain reaction (PCR), next-generation sequencing (NGS) of sputum or bronchoalveolar lavage fluid (BALF), and testing for serum-specific antigens and antibodies. The selection of anti-infective agents should be targeted against these specific pathogens or based on pathogenetic findings to determine appropriate treatment.

### 9.8. Acetylcysteine

A study reported that the combination of budesonide nebulization, azithromycin, montelukast, and oral acetylcysteine (BAMA) effectively alleviated clinical signs and symptoms of PIBO in children, improved pulmonary function and HRCT findings, and reduced the reliance on systemic corticosteroids [24]. This suggests that mucolytic agents may be pharmacologically beneficial in PIBO. However, it is important to note that this was a single-center study with a limited sample size; thus, further validation through larger datasets is necessary.

### 9.9. Gammaglobulin (Intravenous Immunoglobulin (IVIG))

IVIG can be utilized for infection control during the acute phase of PIBO [77]. Yilmaz et al. demonstrated that IVIG not only improved clinical symptoms but also mitigated imaging changes [90].

### 9.10. Bronchoalveolar Lavage

The efficacy of bronchoalveolar lavage remains uncertain, and it is generally not recommended as a routine treatment for PIBO. Theoretically, early lavage may reduce airway inflammatory mediators and inflammatory cells while facilitating the removal of necrotic cells and mucus, which could be beneficial in the early stages of PIBO [91].

### 9.11. Nutritional Support

Children with BO (PIBO) exhibit increased energy expenditure and require adequate caloric intake and energy support to ensure normal growth, development, and immune function, as well as to mitigate the risk of recurrent respiratory infections [1].

### 9.12. Vaccination

Infections in children with PIBO may lead to exacerbations of the disease; therefore, vaccination is essential for the protection of these patients [2]. Children with PIBO during nonacute exacerbation periods should be evaluated to determine the feasibility of vaccination.

All the treatment methods proposed in this work have not been fully validated for efficacy and safety through double-blind controlled trials. Consequently, it is important to clarify that these recommendations are based on a lower level of evidence within the PIBO.

### 9.13. Other Therapies

Tumor necrosis factor inhibitors, antifibrotic therapies, anti-IL-6 therapies, extracorporeal photopheresis, mesenchymal stem cell therapy, and lung transplantation are primarily utilized in children with BOS [1]. The efficacy of these treatments in children with PIBO remains uncertain, despite the shared pathological manifestations. There is insufficient evidence to demonstrate that these treatments are effective for PIBO. PIBO does not progress in its later stages; consequently, lung transplantation is rarely performed [49], and such cases have not been reported on a national or international scale.

## 10. Prognosis

The prognosis for PIBO is generally favorable compared to other forms of BO, with the exception of a few cases resulting in death following infection. A 12-year follow-up study on pulmonary function revealed a gradual improvement in obstructive ventilatory dysfunction, with 65%–80% of PIBO patients showing symptom improvement within one year [49]. In another study, 114 children with PIBO were clinically evaluated and monitored via pulmonary CT after treatment, with a follow-up duration ranging from 6 to 110 months (median 24 months). Clinical symptoms and signs improved in 82.5% of these children, and some also showed improvements in bronchiectasis, atelectasis, hyperinflation, and air trapping [13]. These improvements may be attributed to normal lung development [92].

Children with PIBO had lower quality of life scores than normal children, especially in the health and school domains, with smaller differences in the emotional and social domains [93]. In addition to physiological function limitations such as decreased exercise ability and exercise tolerance, the impact of PIBO on children's health-related quality of life is also reflected in the onset of disease, treatment-related distress, psychological and sleep problems, and other aspects [64]. These influencing factors are intertwined and together reduce children's health-related quality of life. The results of this study remind pediatricians to pay attention to the important significance of quality of life in PIBO, and the impact of PIBO on children's lives cannot be ignored. It is important to strengthen pulmonary rehabilitation programs for children with PIBO to reduce the long-term effects of the disease and improve the quality of life and perception of these patients.

## 11. Future Research Directions

An international consortium of clinical and scientific experts in PIBO undertook a multidisciplinary initiative to develop novel collaborative research methodologies. The investigation concentrated on the following areas.

### 11.1. Good Animal Models of PIBO

There is a notable deficiency of reliable, reproducible, relatively rapid, and cost-effective animal models for PIBO. Current rat models for PIBO are predominantly established through the inhalation of potent chemical agents or lung transplantation. Recent studies have demonstrated that a mouse model of occlusive bronchiolitis can be induced within 28 days using 5% nitric acid aerosol inhalation for 3 hours, thereby providing a simple, economical, and reproducible model for BO research [94].

It has been demonstrated that levels of matrix metalloproteinase-2 (MMP-2), matrix metalloproteinase-9 (MMP-9), IL-6, and TGF-β were significantly elevated, along with an increase in the numbers of leukocytes, neutrophils, and lymphocytes in the lung tissues and BALF of mice with BO. Furthermore, the upregulation of p14ARF reversed these trends in the aforementioned indices and improved both inflammatory responses and airway remodeling in the BO mouse model [95].

The aforementioned studies comprehensively illustrate the advantages of establishing robust animal models, which can elucidate biomarkers and histological changes, guide the formulation of appropriate research targets, and potentially facilitate targeted drug therapies to predict the increased risk of PIBO in at-risk patients or to treat those who have already developed PIBO.

However, there are also notable disadvantages. Although the final pathology of various forms of BO is consistent, there is insufficient evidence to suggest that the pathogenesis, airway inflammatory responses, and fibrotic processes associated with different etiologies of BO are identical. Furthermore, variations in cellular responses, cytokine profiles, and inflammatory mediator changes have been observed across different species. It remains unclear whether animal models prepared by different methods can accurately replicate and reproduce the pathophysiological and immune processes observed in PIBO. Thus, improved models for PIBO remain necessary.

### 11.2. Predictive Biomarkers

Effective molecular markers are crucial for early diagnosis and treatment guidance. We compiled a subset of biomarkers from existing literature; however, due to the low prevalence of PIBO, small sample sizes within individual centers, a limited number of biomarkers assessed in the studies, and variations in detected biomarkers across different geographic regions, researchers have yet to reach a consensus.

A differential assessment of the pathogenesis and biomarkers associated with BO of varying etiologies is essential. BO, as a pathological diagnosis, reflects a common pathological outcome resulting from different underlying causes. In studies examining the potential biomarker KL-6, an increase has been observed in cases of BOS [96], while a decrease was noted in PIBO [40]. Currently, there is insufficient evidence to support that the pathogenesis and biomarkers across different etiologies of BO are identical; thus, further research is necessary for comprehensive cross-sectional assessments.

### 11.3. Nomogram Prediction Models

To investigate objective indicators of PIBO formation following mechanical ventilation, Peng et al. employed logistic regression analysis to summarize 46 cases of PIBO among 863 cases of acute respiratory distress syndrome (ARDS) for the development of a nomogram prediction model [97]. Similarly, Yan et al. utilized an analogous approach to create an early prediction model based on 78 cases of PIBO complicated by 228 instances of severe pneumonia [98]. Both studies were conducted in China and included fewer than 100 children with PIBO, selecting different risk factors; however, they provide optimism for future predictive modeling. Additionally, other predictive models have been reported [99]. It can be posited that if analyses are performed using a large multicenter sample size, early intervention and treatment for PIBO will no longer pose significant challenges.

### 11.4. MicroRNA (miRNA)

miRNAs play a crucial role in the pathogenesis of various respiratory diseases and are central to the epigenetic modification of gene expression, making them potential biomarkers for PIBO [100, 101]. Duecker et al. demonstrated that miR-335-5p, miR-186-5p, miR-30b-5p, and miR-30c-5p influence regulatory pathways associated with inflammation and fibrosis in PIBO [41]. Another study indicated that miR-2, miR-155, miR-21, miR29a, miR-191, and miR-103 serve as biological markers for BOS [102]. To further elucidate the impact of each individual miRNA on PIBO and to enable precise regulation for treatment guidance, data from multicenter studies with large sample sizes are essential.

### 11.5. Microbiome Analysis

It has been proposed that certain microorganisms play a critical role in the development of a healthy immune response; conversely, dysregulated microbial ecology can contribute to chronic inflammatory lung diseases such as asthma, chronic obstructive pulmonary disease (COPD), and CF [103]. Although microbiome data specific to PIBO are currently insufficient, this area presents a promising research direction for understanding the microbial composition in relation to PIBO-related inflammation from a pathophysiological perspective.

### 11.6. Whole Genome Sequencing Study

Given that PIBO is relatively rare and often occurs in conjunction with common respiratory infections, it is plausible that gene–environment interactions contribute to the disease's development. Access to whole exome and potentially whole genome sequencing studies in children experiencing severe lower respiratory tract infections may facilitate the identification of dysregulated genes implicated in PIBO pathology.

The body of research on genetic factors related to PIBO is limited. Teper et al. reported an increased frequency of the HLA-DR8-DQB1-0302 haplotype within the Amerindian PIBO population, which may account for the elevated prevalence of BO in South American countries [23]. There is a pressing need for more comprehensive genetic studies to elucidate the underlying mechanisms of PIBO.

As awareness and attention to PIBO increase, the number of reported cases has risen in recent years. However, due to the limited sample sizes inherent in single-center studies, epidemiological data remain insufficient, and high-quality clinical research on the pathogenesis and treatment of this condition is still lacking. Future efforts should focus on conducting multicenter clinical trials with larger sample sizes while enhancing investigations in molecular biology, pathology, and immunology to provide a scientific foundation for early intervention and improved prognostic outcomes.

## Figures and Tables

**Table 1:** Infections.

Adenovirus 3, 7, 11, 21, and 55
*Mycoplasma pneumoniae*
Influenza
Parainfluenza
Measles
Respiratory syncytial virus (RSV)
Herpes simplex virus (HSV)
Varicella
Human metapneumovirus
*Bordetella pertussis*
Tuberculosis
*Chlamydia pneumoniae*
Legionella
COVID-19

**Table 2:** PIBO biomarker.

Parameter	Serum	BAL/sputum	Evidence	Reference
Neutrophils	—	Increased	A	Eckrich et al. [36]
IL-1β	—	Increased	A	Rosewich et al. [37]
IL-6	—	Increased	A	Rosewich et al. [37]
IL-8	—	Increased	A	Koh et al. [38]
IL-18	Decreased	—	B	Eyüboğlu et al. [17]
YKL-40	Increased	—	B	Jang et al. [39]
KL-6	Decreased	—	B	Bayhan et al. [40]
miR-335-5p/miR-186-5p/miR-30b-5p/miR-30c-5p	Decreased	—	B	Duecker et al. [41]
Caspase-1	Increased	—	B	Eyüboğlu et al. [17]
TGF-β	—	Increased	B	Duecker et al. [41] Kang et al. [42]
Postn	—	Increased	B	Kang et al. [42]
β2-defensin	Increased	—	B	Gedik et al. [43]
IFN-γ	Increased	—	B	Hodge et al. [44]
Cathelicidin (LL-37)	Increased	—	B	Gedik et al. [43]

**Table 3:** BO score.

Predictor variable	Value	
	Present	Absent
Typical clinical history	4	0
Adenovirus history	3	0
Mosaic pattern in HRCT	4	0

*Note:* Score range 0–11.

## Data Availability

This review article synthesizes existing literature and does not generate new experimental datasets. All referenced data sources are properly cited and publicly available through their original publications.
